# The maladaptive domains according to the alternative model of personality disorders (AMPD) criterion B in patients with affective disorders and temperamental triads related to these domains: two unique profiles

**DOI:** 10.1186/s40359-023-01122-5

**Published:** 2023-03-28

**Authors:** Saeid Komasi, Minoo Jananeh, Sahar Mahdavi, Tahereh Shademan, Anis Vaysi, Mehrnoosh Shahlaee, Atefeh Mirani, Zahra Chamandoust, Mozhgan Saeidi

**Affiliations:** 1 Department of Neuroscience and Psychopathology Research, Mind GPS Institute, Kermanshah, Iran; 2 Department of Psychology, University of Kurdistan, Sanandaj, Iran

**Keywords:** Affective disorder, Bipolar mood, Major depression, Temperament, Personality pathology

## Abstract

**Objectives:**

The study aimed to (*i*) compare the maladaptive domains and facets according to the Alternative Model of Personality Disorders (AMPD) Criterion B in patients with a type II bipolar disorder (BD-II) or major depressive disorder (MDD) with healthy controls (HCs), and (*ii*) investigating the relationship between affective temperaments and these domains and facets in the total sample.

**Methods:**

Outpatients diagnosed with current BD-II (n = 37; female 62.2%) or MDD (n = 17; female 82.4%) based on the fifth edition of the Diagnostic and Statistical Manual of Mental Disorders (DSM-5) criteria and community HCs (n = 177; female 62.1%) in Kermanshah from July to October 2020 included this case-control study. All participants completed the Personality Inventory for DSM-5 (PID-5), the Temperament Evaluation of Memphis, Pisa, Paris, and San Diego Autoquestionnaire (TEMPS-A), and the second version of the Beck Depression Inventory (BDI-II). Data were analyzed using analysis of variance (ANOVA), Pearson correlation, and multiple regression.

**Results:**

The score of patients with BD-II in all five domains and those with MDD in three domains including negative affectivity, detachment, and disinhibition are significantly higher than the HCs (*p* < 0.05). Depressive temperament (related to negative affectivity, detachment, and disinhibition) and cyclothymic temperament (related to antagonism and psychoticism) were the most important correlates of the maladaptive domains.

**Conclusions:**

Two unique profiles are proposed, including three domains of negative affectivity, detachment, and disinhibition associated with the depressive temperament for MDD, and two domains of antagonism and psychoticism related to cyclothymic temperament for BD-II.

## Introduction

The dimensional model of psychopathology has been proposed in the fifth edition of the Diagnostic and Statistical Manual of Mental Disorders (DSM-5) over the last decade to overcome the limitations and challenges of traditional classification systems [1]. Although DSM-5 has significant revisions and improvements compared to previous versions, more fundamental revisions, especially in the field of personality disorders, are presented in the third section in the form of the Alternative Model of Personality Disorders (AMPD) [2, 3]. In this section, in addition to eliminating four diagnostic categories including paranoid, schizoid, histrionic, and dependent personality disorders, the importance of 25 maladaptive facets and five major domains of maladaptive personality are highlighted [1, 4]. According to Criterion B in AMPD, maladaptive domains include negative affectivity, detachment, antagonism, disinhibition, and psychoticism [5]. Criterion A includes intrapersonal (identity and self-direction) and interpersonal (empathy and intimacy) functioning [6]. AMPD, which is based on factor analysis methods, was initially proposed solely to reconsideration of personality disorders in previous versions of the DSM [1]. Nevertheless, this model was extended to the whole field of psychopathology by Kotov et al. [7, 8]. The Hierarchical Taxonomy of Psychopathology (HiTOP) introduces many diagnostic categories of psychopathology in traditional classification systems as subsets of five major maladaptive domains called higher-order spectra [8, 9]. However, this factor analysis model still faces challenges for some diagnostic categories such as somatoform and bipolar spectrum disorders [8, 10–12].

Bipolar spectrum/affective disorders include depression with or without periods of hypomania/mania [13]. According to the results of several studies, the lifetime prevalence of these disorders is reported between 1 and 2.4% in the world [14, 15] and 0.1 to 8.3% in Iran [16–18]. Affective disorders are associated with many health consequences and outcomes, including alcoholism [19], substance abuse [20], aggression [21], and suicide ideations and attempts [22, 23]. From 1990 to 2013, the prevalence of bipolar disorder increased by 49%, leading to approximately 10 million years of disability-adjusted life years (DALYs) [24]. Meanwhile, from 1990 to 2017, DALYs associated with bipolar disorder increased by 54.4% [25].

In general, affective disorders are one of the ten most costly diseases in the USA [26] that impose a significant annual financial burden on the economies and health systems of countries [27]. This situation highlights the importance of paying attention to diagnostic and therapeutic models associated with affective disorders. In recent years, the role of some genetic [28] and environmental risk factors [29], family and heredity [30], and personality models including the big five theory [31], temperament and character theory [32], and affective temperaments [33] have been repeatedly examined. In particular, studies have confirmed the complex and stable relationships between affective temperaments proposed by Akiskal et al. [34] and affective disorders [33, 35]. The affective temperaments include depressive, cyclothymic, hyperthymic, irritable, and anxious temperaments [34]. Despite the importance of affective temperaments in affective disorders, our search for access to studies examining the relationship between these temperaments and the proposed domains by AMPD was fruitless. Based on these considerations, the present study was conducted to (*i*) compare the maladaptive domains and facets between the three groups of patients with type II bipolar disorder (BD-II), patients with major depressive disorder (MDD), and healthy controls (HCs), and (*ii*) the relationship between each of the five affective temperaments and the five maladaptive domains.

## Methods

### Study design, participants, and data collection

Outpatients diagnosed with current BD-II (n = 37; female 62.2%) or MDD (n = 17; female 82.4%) referred to a psychology outpatient clinic in Kermanshah city from July to October 2020 entered this case-control study consecutively. Our clinic is a small non-governmental psychotherapy center for outpatients, which is mainly visited by the population with subclinical neurotic symptoms. Psychiatric comorbidities including obsessive-compulsive disorder, generalized anxiety disorder, social anxiety disorder, panic disorder, and somatic symptom and related disorders in patients with affective disorders were also screened by two expert clinical psychologists. These affective disorders and comorbidities were diagnosed using a clinical interview according to DSM-5 criteria. Inclusion criteria were 18 to 60 years old, the affective disorder first diagnosed, five or more years of education, and fluency in the Farsi language. Also, all patients were free from any type of psychotherapy and psychiatric medications in the last six months, active substance abuse or addiction, and chronic neurological diseases such as multiple sclerosis. The patients with affective disorders due to another medical condition and substance/medication-induced affective disorders were also excluded. HCs included 177 (female 62.1%) people in the community without any history of psychiatry. The initial sample consisted of 238 people, 6 of whom were excluded due to drug addiction or a history of psychiatry and current medication or psychotherapy. Because of the possibility of MDD, 55 people with a score of 20 or higher on the Beck Depression Inventory (BDI-II) were excluded. After completing the self-report form for demographic information (age, gender, education level, job, marital status, and psychiatric history), all participants answered the Persian long form of the Personality Inventory for DSM-5 (PID-5; 220 items), the Persian short form of the Temperament Evaluation of Memphis, Pisa, Paris, and San Diego Autoquestionnaire (TEMPS-A; 35 items), and the Persian version of BDI-II (21 items).

### Data measurement

#### Personality inventory for DSM-5 (PID-5)

This 220-item self-report inventory was developed by Krueger et al. (2013) to assess the five maladaptive domains and 25 facets according to criterion B of the AMPD proposed in DSM-5 Section-III [4]. The domains and facets are included negative affectivity (emotional liability, anxiousness, and separation insecurity), detachment (withdrawal, anhedonia, and intimacy avoidance), antagonism (manipulativeness, deceitfulness, and grandiosity), disinhibition (irresponsibility, impulsivity, and distractibility), and psychoticism (unusual beliefs & experiences, eccentricity, and perceptual dysregulation). Other facets are included attention-seeking, callousness, depressivity, hostility, perseveration, restricted affectivity, rigid perfectionism, risk-taking, submissiveness, and suspiciousness. Items response is based on a Likert scale ranging from 0 to 3 [4]. Hemmati et al. (2019) confirmed Cronbach’s alphas of the Persian version for the PID-5 domains: disinhibition (0.89), detachment and negative affectivity (0.93), and antagonism and psychoticism (0.94). Also, Cronbach’s alphas for the 25 trait facets were acceptable, ranging from 0.70 to 0.94 [36].

#### Temperament evaluation of Memphis, Pisa, Paris, and San Diego autoquestionnaire (TEMPS-A)

The Persian version of the TEMPS-A questionnaire includes 35 questions (Yes/No) in five subscales including depressive (items 1–6, 9, 10), cyclothymic (items 7, 8, 11, 12, 17, 18, 27) hyperthymic (items 13–16 and 19–22), irritable (items 23, 25, 32–35), and anxious (items 24, 26, 28–31) temperaments. Khalili et al. (2018) confirmed the reliability and validity of this tool in the Iranian sample [37].

#### Beck depression inventory (BDI-II)

This 21-item questionnaire was designed by Beck et al. [38]. The score of each item is between 0 and 3 and the total score varies from 0 to 63. According to Beck et al. [38] a cut point of 20 or higher indicates moderate to severe depression. The reliability and validity of the Persian version of this questionnaire have been confirmed [39].

#### Data analysis

In the first stage, sociodemographic data including age, gender, education level, job, marital status, depression, and psychiatric comorbidities were compared between the three groups using the chi-square test for discontinuous variables and analysis of variance (ANOVA) and Tukey post hoc test for continuous variables including age and depression assessed using BDI-II. In the main analysis and after confirming the non-violation of statistical assumptions, ANOVA and Tukey post hoc test was used for comparing the maladaptive domains and facets of the AMPD between the three groups. Then, the Pearson correlation coefficient was used to investigate the correlations between affective temperaments assessed by TEMPS-A and maladaptive domains and facets in the total sample. In addition, the effect sizes (correlations) were reported following Cohen [40]. According to Cohen, there are present significant correlations in five categories: effect size (*r*) < 0.30 (small, one symbol), < 0.50 (medium, two symbols), < 0.70 (large, three symbols), and ≥ 0.70 (very large, four symbols). Finally, five separate multiple regression analyzes (ENTER method) were performed to predict the maladaptive domains by affective temperaments. Temperamental triads associated with maladaptive domains were also displayed in the form of a figure. All analyses were performed using the twentieth version of SPSS software and a *p* < 0.05 was considered as the significance level.

## Results

Table 1 shows the sociodemographic information in two clinical groups include BD-II and MDD and HCs. As can be seen, there is no significant difference in gender, age, and job status between the cases and HCs. However, there is a significant difference between the three groups in other variables (*p* < 0.05).

### Compare the maladaptive domains and facets between the cases and healthy controls

Table 2 shows the differences between groups in maladaptive domains and facets. The scores of patients with BD-II in all five domains are significantly higher than the HCs. Patients with MDD also scored significantly higher than the HCs in three domains of negative affectivity, detachment, and disinhibition (*p* < 0.05). The results of this table also show that there is a significant difference between the three groups in 19 maladaptive facets (*p* < 0.05).

### The relationship between affective temperaments and the maladaptive domains

Table 3 shows the correlations between affective temperaments and maladaptive domains and facets in the total sample. Stronger correlations are seen between the three depressive, cyclothymic, and irritable temperaments compared to hyperthymic and anxious temperaments with maladaptive domains. This table also shows a wide range of significant correlations between affective temperaments and maladaptive facets (*p* < 0.05).

**Table 1:** The comparison of sociodemographic data among cases and HCs

Socio-demographics	BD-II (n = 37)	MDD (n = 17)	HCs (n = 177)	*P* value
Age (Mean ± SD)	28.8 ± 8.1	35.6 ± 10.5	31.5 ± 7.7	0.015 ^a^
Gender, female (%)	23 (62.2)	14 (82.4)	110 (62.1)	0.249 ^b^
Marital status (%)				0.001 ^b^
Single	26 (70.3)	6 (35.3)	78 (44.1)	
Marriage	8 (21.6)	9 (52.9)	94 (53.1)	
Widow/divorced	3 (8.1)	2 (11.8)	5 (2.8)	
Education level (%)				0.001 ^b^
Under diploma	2 (5.4)	6 (35.3)	6 (3.4)	
Diploma	9 (24.3)	3 (17.6)	47 (26.6)	
Academic	26 (70.3)	8 (47.1)	124 (70)	
Job (%)				0.085 ^b^
College student	8 (21.6)	4	35 (19.8)	
Employed	5 (13.5)	1	38 (21.5)	
Self-employed	14 (37.9)	3	37 (20.9)	
Housekeeper	3 (8.1)	7	44 (24.8)	
Other	7 (18.9)	2	23 (13)	
Psychiatric comorbidity (%)				
Obsessive-Compulsive Disorder	1 (2.7)	2 (11.8)	0 (0)	0.001 ^b^
Generalized Anxiety Disorder	0 (0)	2 (11.8)	0 (0)	0.001 ^b^
Social Anxiety Disorder	4 (10.8)	2 (11.8)	0 (0)	0.001 ^b^
Panic Attack	2 (5.4)	2 (11.8)	0 (0)	0.001 ^b^
Somatoform disorder	3 (8.1)	6 (35.3)	0 (0)	0.001 ^b^
Depression (Mean ± SD)	23.66 ± 12.58	32.59 ± 12.29	8.03 ± 5.37	0.001 ^c^

Abbreviation: BD-II = bipolar disorder; MDD = major depression disorder; HCs = healthy controls; a = ANOVA, Tukey post hoc test: MDD > BD-II; b = Chi-square test; c = ANOVA, Tukey post hoc test: MDD > BD-II > HC.

**Table 2:** The comparison of the maladaptive domains and facets among cases and HCs

Maladaptive domains and facets (Mean ± SD)	BD-II (n = 37)	MDD (n = 17)	HCs (n = 177)	Differences (ANOVA, Tukey post hoc) ^a^
*Negative Affectivity*	32.44 ± 13.84	38.29 ± 11.80	19.71 ± 9.72	BD-II = MDD > HC
Emotional liability	11.98 ± 4.77	10.59 ± 4.69	6.92 ± 3.98	BD-II = MDD > HC
Anxiousness	13.11 ± 7.06	19.41 ± 5.63	7.10 ± 4.50	MDD > BD-II > HC
Separation insecurity	7.35 ± 4.80	8.29 ± 4.73	5.69 ± 4.02	MDD > HC
*Detachment*	27.29 ± 13.75	30.23 ± 11.00	17.07 ± 9.55	BD-II = MDD > HC
Withdrawal	11.92 ± 7.58	10.64 ± 5.73	6.64 ± 4.87	BD-II = MDD > HC
Anhedonia	10.24 ± 5.60	14.41 ± 5.68	5.69 ± 3.46	MDD > BD-II > HC
Intimacy avoidance	5.13 ± 3.99	5.18 ± 3.50	4.73 ± 3.56	ns
*Antagonism*	22.28 ± 9.04	15.35 ± 7.54	17.58 ± 10.19	BD-II > MDD = HC
Manipulativeness	4.22 ± 2.22	3.20 ± 2.26	4.14 ± 2.75	ns
Deceitfulness	8.90 ± 5.86	6.56 ± 4.92	6.58 ± 5.52	ns
Grandiosity	9.16 ± 3.74	5.59 ± 3.64	6.87 ± 3.51	BD-II > MDD = HC
*Disinhibition*	25.09 ± 11.54	24.64 ± 10.97	14.51 ± 10.08	BD-II = MDD > HC
Irresponsibility	5.11 ± 3.22	5.53 ± 3.50	3.95 ± 3.54	ns
Impulsivity	6.98 ± 4.41	6.06 ± 5.16	3.98 ± 3.94	BD-II > HC
Distractibility	25.09 ± 11.54	24.64 ± 10.97	14.51 ± 10.08	BD-II = MDD > HC
*Psychoticism*	32.49 ± 17.01	27.70 ± 18.75	18.99 ± 14.63	BD-II > HC
Unusual Beliefs	7.38 ± 4.69	5.69 ± 4.09	5.05 ± 3.94	BD-II > HC
Eccentricity	14.17 ± 9.39	11.57 ± 10.48	8.05 ± 7.92	BD-II > HC
Perceptual Dysregulation	10.95 ± 5.88	10.44 ± 7.49	5.89 4.64	BD-II = MDD > HC
*Other facets*				
Attention Seeking	13.51 ± 4.91	9.26 ± 5.67	9.77 ± 5.11	BD-II > MDD = HC
Callousness	9.06 ± 5.47	8.41 ± 8.25	7.38 ± 6.49	ns
Depressivity	15.13 ± 11.14	21.23 ± 10.59	5.34 ± 5.51	MDD > BD-II > HC
Hostility	15.98 ± 5.95	13.71 ± 5.79	9.18 ± 5.45	BD-II = MDD > HC
Perseveration	11.86 ± 5.88	12.83 ± 5.36	7.87 ± 4.60	BD-II = MDD > HC
Restricted Affectivity	8.24 ± 4.13	6.64 ± 3.05	5.92 ± 3.42	BD-II > HC
Rigid Perfectionism	17.60 ± 4.92	14.63 ± 5.42	12.58 ± 5.25	BD-II > HC
Risk Taking	20.86 ± 6.78	17.23 ± 7.34	18.74 ± 7.16	ns
Submissiveness	5.32 ± 2.30	4.00 ± 2.64	4.07 ± 2.29	BD-II > HC
Suspiciousness	10.59 ± 4.54	9.70 ± 2.91	7.92 ± 3.06	BD-II > HC

Abbreviation: BD-II = bipolar disorder; MDD = major depression disorder; HCs = healthy controls; a = *p* value < 0.05.

**Table 3:** The correlations between variables in the total sample (n = 231)

Maladaptive domains and facets	Depressive			Cyclothymic			Hyperthymic			Irritable			Anxious		
	*r*	*p*	*ES* ^*a*^	*r*	*p*	*ES* ^*a*^	*r*	*p*	*ES* ^*a*^	*r*	*p*	*ES* ^*a*^	*r*	*p*	*ES* ^*a*^
*Negative Affectivity*	0.669	0.001	↑↑↑	0.507	0.001	↑↑↑	− 0.153	0.020	↓	0.658	0.001	↑↑↑	0.463	0.001	↑↑
Emotional liability	0.488	0.001	↑↑	0.553	0.001	↑↑↑	0.057	0.392		0.426	0.001	↑↑	0.336	0.001	↑↑
Anxiousness	0.685	0.001	↑↑↑	0.389	0.001	↑↑	− 0.306	0.001	↓↓	0.699	0.001	↑↑↑	0.450	0.001	↑↑
Separation insecurity	0.402	0.001	↑↑	0.298	0.001	↑	− 0.055	0.406		0.419	0.001	↑↑	0.316	0.001	↑↑
*Detachment*	0.521	0.001	↑↑↑	0.217	0.001	↑	− 0.347	0.001	↓↓	0.474	0.001	↑↑	0.363	0.001	↑↑
Withdrawal	0.419	0.001	↑↑	0.175	0.008	↑	− 0.182	0.006	↓	0.364	0.001	↑↑	0.269	0.001	↑
Anhedonia	0.686	0.001	↑↑↑	0.290	0.001	↑	− 0.443	0.001	↓↓	0.639	0.001	↑↑↑	0.445	0.001	↑↑
Intimacy avoidance	0.055	0.402		0.015	0.815		− 0.213	0.001	↓	0.061	0.356		0.120	0.068	
*Antagonism*	0.234	0.001	↑	0.462	0.001	↑↑	0.309	0.001	↑↑	0.237	0.001	↑	0.347	0.001	↑↑
Manipulativeness	0.084	0.205		0.333	0.001	↑↑	0.308	0.001	↑↑	0.120	0.069		0.278	0.001	↑
Deceitfulness	0.308	0.001	↑↑	0.463	0.001	↑↑	0.137	0.037	↑	0.302	0.001	↑↑	0.376	0.001	↑↑
Grandiosity	0.107	0.103		0.314	0.001	↑↑	0.410	0.001	↑↑	0.100	0.128		0.173	0.009	↑
*Disinhibition*	0.644	0.001	↑↑↑	0.503	0.001	↑↑↑	− 0.132	0.046	↓	0.532	0.001	↑↑↑	0.488	0.001	↑↑
Irresponsibility	0.392	0.001	↑↑	0.355	0.001	↑↑	− 0.024	0.714		0.313	0.001	↑↑	0.345	0.001	↑↑
Impulsivity	0.514	0.001	↑↑↑	0.405	0.001	↑↑	− 0.027	0.683		0.432	0.001	↑↑	0.468	0.001	↑↑
Distractibility	0.653	0.001	↑↑↑	0.478	0.001	↑↑	− 0.228	0.001	↓	0.542	0.001	↑↑↑	0.407	0.001	↑↑
*Psychoticism*	0.373	0.001	↑↑	0.480	0.001	↑↑	0.076	0.248		0.392	0.001	↑↑	0.362	0.001	↑↑
Unusual Beliefs	0.135	0.040	↑	0.283	0.001	↑	0.110	0.096		0.211	0.001	↑	0.154	0.019	↑
Eccentricity	0.319	0.001	↑↑	0.457	0.001	↑↑	0.100	0.129		0.343	0.001	↑↑	0.344	0.001	↑↑
Perceptual Dysregulation	0.493	0.001	↑↑	0.479	0.001	↑↑	− 0.016	0.804		0.454	0.001	↑↑	0.408	0.001	↑↑
*Other facets*															
Attention Seeking	0.257	0.001	↑	0.432	0.001	↑↑	0.239	0.001	↑	0.249	0.001	↑	0.260	0.001	↑
Callousness	0.325	0.001	↑↑	0.336	0.001	↑↑	0.051	0.439		0.334	0.001	↑↑	0.439	0.001	↑↑
Depressivity	0.694	0.001	↑↑↑	0.406	0.001	↑↑	− 0.294	0.001	↓	0.626	0.001	↑↑↑	0.426	0.001	↑↑
Hostility	0.555	0.001	↑↑↑	0.507	0.001	↑↑↑	− 0.033	0.616		0.564	0.001	↑↑↑	0.477	0.001	↑↑
Perseveration	0.526	0.001	↑↑↑	0.408	0.001	↑↑	− 0.107	0.103		0.435	0.001	↑↑	0.381	0.001	↑↑
Restricted Affectivity	0.237	0.001	↑	0.201	0.002	↑	0.004	0.956		0.167	0.011	↑	0.142	0.031	↑
Rigid Perfectionism	0.327	0.001	↑↑	0.399	0.001	↑↑	0.057	0.390		0.340	0.001	↑↑	0.274	0.001	↑
Risk Taking	0.052	0.432		0.358	0.001	↑↑	0.254	0.001	↑	0.071	0.282		0.182	0.006	↑
Submissiveness	0.329	0.001	↑↑	0.213	0.001	↑	− 0.150	0.023	↓	0.219	0.001	↑	0.163	0.013	↑
Suspiciousness	0.407	0.001	↑↑	0.376	0.001	↑↑	− 0.027	0.680		0.401	0.001	↑↑	0.412	0.001	↑↑

Abbreviation: ES = effect size; a = significant effect size (r) < 0.30 (small, one symbol), < 0.50 (medium, two symbols), < 0.70 (large, three symbols), ≥ 0.70 (very large, four symbols).

Table 4 shows the results of multiple regression analysis for predicting maladaptive domains in the total sample. The largest effect size associated with negative affectivity (*β* = 0.321, *p* < 0.001), detachment (*β* = 0.267, *p* = 0.004), and disinhibition (*β* = 0.443, *p* < 0.001) was depressive temperament; while the biggest effect size related to antagonism (*β* = 0.310, *p* < 0.001) and psychoticism (*β* = 0.351, *p* < 0.001) was cyclothymic temperament. Four models including five predictive temperaments were able to explain 53.1%, 34%, 30.9%, and 47.1% of the variance of negative affectivity, detachment, antagonism, and disinhibition, respectively. The regression model associated with the psychoticism domain consisted of four temperaments and the hyperthymic temperament was excluded due to a lack of correlation with this maladaptive domain. This model was also able to significantly explain 27.3% of the variance of psychoticism (*p* < 0.001).

### The driven model

**Fig. 1:**
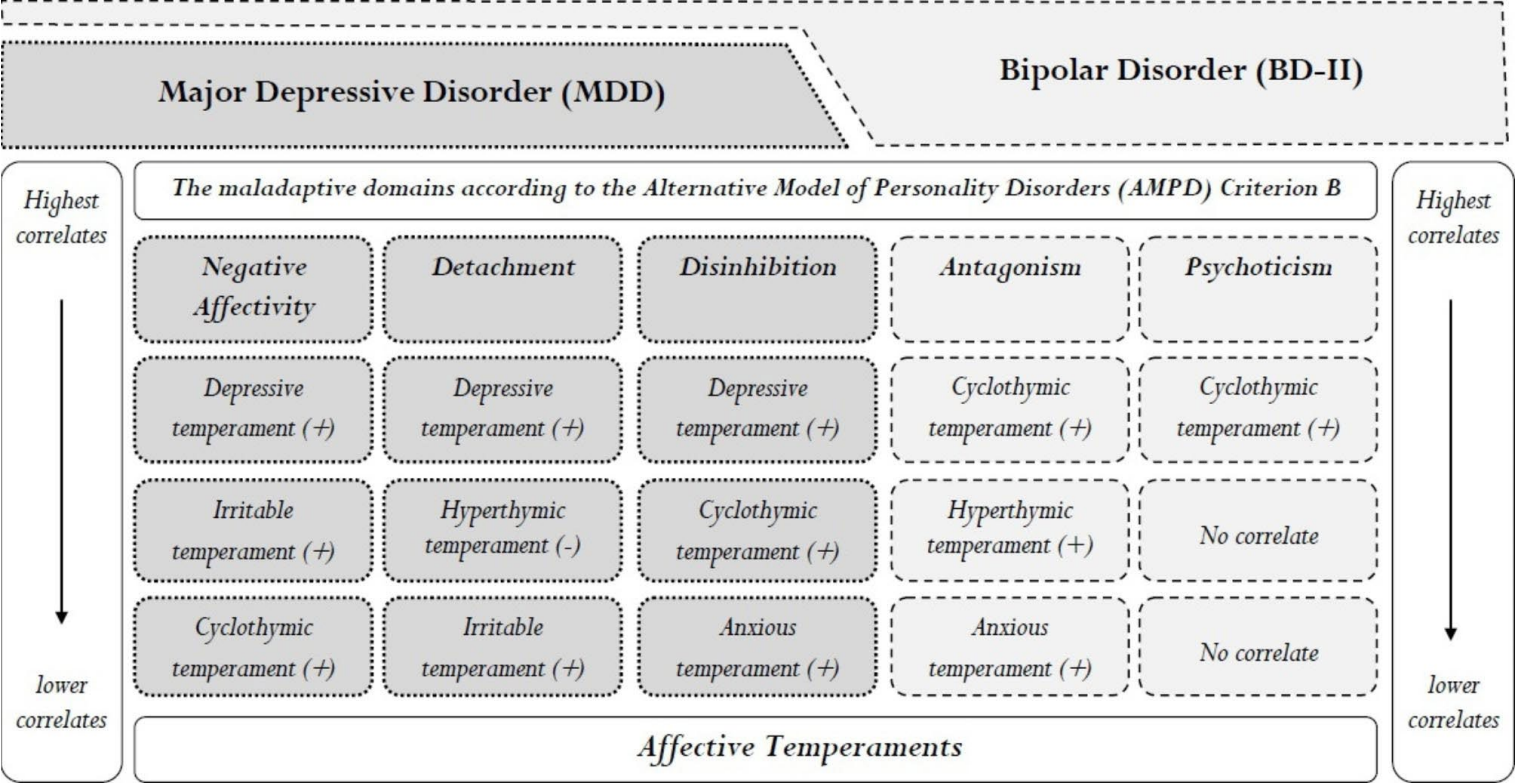
Temperamental triads related to the maladaptive domains according to the AMPD Criterion B in the total sample

The temperamental triads related to the maladaptive domains according to the AMPD Criterion B in the total sample can be seen in Fig. 1. This figure shows that depressive and cyclothymic temperaments are the most important correlates of all maladaptive domains. However, the combination of these temperaments with other temperaments in explaining the maladaptive domains is different and unique.

**Table 4:** The multiple regression analysis for predicting maladaptive domains in the total sample

*Negative Affectivity*	*B*	*β*	*t*	*p*	Summary of the model
Depressive	1.723	0.321	4.185	0.001	*R* = 0.728 *R*^*2*^ = 0.531 * F* = 50.884 *P* < 0.001
Cyclothymic	1.530	0.204	3.501	0.001	
Hyperthymic	− 0.308	− 0.048	− 0.939	0.349	
Irritable	2.361	0.310	4.250	0.001	
Anxious	0.018	0.002	0.30	0.976	
*Detachment*	B	β	t	*p*	Summary of the model
Depressive	1.330	0.267	2.938	0.004	*R* = 0.583 *R*^*2*^ = 0.340 * F* = 23.227 *P* < 0.001
Cyclothymic	− 0.019	− 0.003	− 0.039	0.969	
Hyperthymic	− 1.398	− 0.237	− 3.882	0.001	
Irritable	1.316	0.186	2.154	0.032	
Anxious	0.810	0.086	1.213	0.226	
*Antagonism*	B	β	t	*p*	Summary of the model
Depressive	0.302	0.069	0.745	0.457	*R* = 0.556 *R*^*2*^ = 0.309 * F* = 20.101 *P* < 0.001
Cyclothymic	1.883	0.310	4.379	0.001	
Hyperthymic	1.430	0.277	4.439	0.001	
Irritable	− 0.315	− 0.051	− 0.577	0.564	
Anxious	1.806	0.219	3.025	0.003	
*Disinhibition*	B	β	t	*p*	Summary of the model
Depressive	2.120	0.443	5.309	0.001	*R* = 0.686 *R*^*2*^ = 0.471 * F* = 40.024 *P* < 0.001
Cyclothymic	1.610	0.235	3.797	0.001	
Hyperthymic	− 0.249	− 0.043	− 0.785	0.434	
Irritable	0.076	0.011	0.141	0.888	
Anxious	1.192	0.128	2.024	0.044	
*Psychoticism*	B	β	t	*p*	Summary of the model
Depressive	0.297	0.042	0.471	0.638	*R* = 0.523 *R*^*2*^ = 0.273 * F* = 21.228 *P* < 0.001
Cyclothymic	3.446	0.351	5.191	0.001	
Hyperthymic	Na	Na	Na	Na	
Irritable	1.214	0.122	1.344	0.180	
Anxious	1.521	0.114	1.544	0.124	

Abbreviation: Na = not applicable.

## Discussion

In the present study, we aimed to compare the maladaptive domains and facets of AMPD [1, 2] between outpatients with BD-II or MDD and HCs. According to the present findings, patients with BD-II in all five maladaptive domains and patients with MDD in the three domains of negative affectivity, detachment, and disinhibition showed higher scores than HCs. These findings are consistent with previous reports that indicate the ability of AMPD to diagnose a variety of affective disorders and their associated risks [41, 42]. Our results also showed that patients with BD-II scored higher in the antagonism domain than patients with MDD. Considering the maladaptive facets of attention-seeking, depressivity, hostility, and perseveration, the scores of patients with affective disorders were higher than the HCs. Compared to HCs, patients with BD-II also reported higher scores on restricted affectivity, rigid perfectionism, submissiveness, and suspiciousness. These findings indicate a more severe psychopathology of BD-II compared to MDD. The results of two studies have reported more severe personality and functional disorders in patients with BD-II [43, 44].

Our other aim was to investigate the relationship between affective temperaments [34] and five maladaptive domains [4] in the whole sample. The results showed that depressive and cyclothymic temperaments are the strongest correlates of maladaptive domains. Depressive temperament was able to significantly explain the variance of the three domains of negative affectivity, detachment, and disinhibition; while the cyclothymic temperament was able to explain the variance of antagonism and psychoticism domains. The findings related to the objectives of the present study are discussed in more detail below.

In the negative affectivity domain, patients with affective disorders reported higher scores than HCs. Patients with MDD in all three facets related to this domain including emotional liability, anxiousness, and separation insecurity, and patients with BD-II in the two facets of emotional liability and anxiousness showed higher scores than the HCs. This finding indicates the importance of this maladaptive domain and its facets in the occurrence and persistence of affective disorders. The significance of this finding is highlighted when it is seen that affective temperaments - especially depressive - also have the highest correlation with this domain and can explain 53% of the variance of negative affectivity. Recent studies have confirmed the role of negative affect [45], anxiety [46], and emotion regulation problems [47] in patients with affective disorders. The results of a review also point to the relationship between affective temperaments - especially depressive temperament - and negative affect/neuroticism [48].

In the detachment domain and the two facets of withdrawal and anhedonia, patients with affective disorders also reported higher scores than HCs. Also, patients with MDD showed higher scores in the anhedonia facet compared to BD-II. These findings may be due to the current hypomania/mania phase of patients with BD-II. Since the anhedonia facet in DSM-5 [1] is a diagnostic criterion for affective disorders, this finding was to be expected. One study also confirmed the association between low mood and sadness with withdrawal [49]. This claim can also be confirmed by the strong relationship between depressive temperament and the detachment domain in the present study. The results of a new study point to the importance of the mediating role of attachment (the opposite pole of detachment) in the relationship between affective temperaments and depressive symptoms [50]. In general, patients with affective disorders have problems with interpersonal functions in addition to intrapersonal functions [51]. Detachment in patients with affective disorders may also be affected by some dopamine receptors affecting the disease [52].

In the antagonism domain, there was no significant difference between patients with MDD and HCs; However, patients with BD-II showed higher scores in this domain and grandiosity facet than HCs and patients with MDD. Grandiosity delusion is an expected sign in the hypomania/mania phase of patients with BD-II and is one of the signs of criterion B in DSM-5 [1, 53]. According to the present results, cyclothymic temperament is the most important correlate of the antagonism domain. The correlation between cyclothymic and hostile personality traits and aggressive behaviors with bipolar spectrum disorders has already been confirmed [54, 55].

In the disinhibition domain and the distractibility facet, patients with affective disorders reported higher scores than HCs. Also, the impulsivity facet in patients with BD-II was higher than HCs. Impulsive behaviors are one of the signs of criterion B in DSM-5 [1] which has already been confirmed [56]. Although according to the findings of the current study, there is a significant difference between patients with MDD and HCs in the disinhibition domain, this difference is mainly due to the distractibility facet. The distractibility facet or lack of concentration is one of the most important symptoms of depression [57]. Conversely, the difference between patients with BD-II and HCs arises simultaneously from the distractibility and impulsivity facets. Consistent with this discussion, we find that depressive and cyclothymic temperaments are the most important correlates of this domain. Previous studies have reported a correlation between these affective temperaments and uninhibited behaviors [58, 59].

In the psychoticism domain and all facets including unusual beliefs and experiences, eccentricity, and perceptual dysregulation, patients with BD-II reported higher scores of HCs. Patients with MDD showed higher scores than HCs only in the maladaptive facet of perceptual dysregulation. The incidence of psychotic symptoms, especially in patients with BD-II-I and emotional and perceptual dysregulation in depressed patients, has been previously reported [47, 60]. Compared to other maladaptive domains, the psychoticism domain was more poorly predicted by affective temperaments. Our results showed that cyclothymic temperament is the only and most important temperamental correlate of this domain. The study by Mahon et al. (2013) pointed to a strong relationship between cyclothymic temperament and psychotic symptoms [61].

Generally, our study is a pioneer in investigating the relationship between affective temperaments and maladaptive domains in patients with affective disorders. Although the present case-control design was able to report maladaptive domains and facets between the three groups of BD-II, MDD, and HCs, some limitations may be raised. The sample size was small in patients with affective disorders, especially patients with MDD, which may increase the bias in the results. Our clinic is a small non-governmental psychotherapy center for outpatients, which mainly populations with subclinical neurotic symptoms refer to it rather than clinical cases. Hence, few patients with MDD attended the clinic during the study period and we were unable to use a larger sample. We reported the correlation between the variables in the whole sample. In the case of access to a larger sample of patients with affective disorders, two separate correlations can be reported in these patients and HCs. This could provide valuable insights for clinicians and new psychopathological classification systems such as HiTOP. Due to the small sample size of patients with affective disorders, we did not examine the co-occurrence of personality disorders and affective disorders. It should be noted that AMPD was first proposed to explain personality disorders and these disorders play an important role in maladaptive domains and facets. Therefore, future studies should examine the prevalence and severity of personality disorders in these patients. We did not match the groups and used a larger control group compared to the case groups to increase statistical power. Although there was no significant difference between the mean age and sex of patients with affective disorders and the HCs, marital status was different between the groups. Some studies have suggested a possible role for marriage and the support system in lower periods of depression and reduced hospitalization [62, 63]. Therefore, matching cases and controls in terms of marital status may be necessary.

## Conclusions

In sum, patients with affective disorders have a more impaired profile than HCs in approximately 80% of maladaptive facets according to AMPD. Patients with BD-II in all five maladaptive domains and patients with MDD in the three domains of negative affectivity, detachment, and disinhibition showed higher scores than HCs. Also, patients with BD-II scored higher in the antagonism domain than patients with MDD. Thus, the maladaptive profile of patients with BD-II is probably more severe than that of depressed patients. Our findings highlight the unique role of the temperamental triads associated with each maladaptive domain. Depressive temperament associated with the three domains of negative affectivity, detachment, and disinhibition, and cyclothymic temperament associated with the two domains of antagonism and psychoticism are the most important correlates of maladaptive domains. In total, two unique profiles are proposed, including three domains of negative affectivity, detachment, and disinhibition associated with depressive temperament for MDD, and two domains of antagonism and psychoticism related to cyclothymic temperament for BD-II. Future studies could examine more complex relationships between maladaptive domains and affective disorders by considering personality disorders.

## Data Availability

The current study data are available on reasonable request to S.K., S_komasi63@yahoo.com.
